# Multivariate dataset on cognitive predictors of Indian consumers' purchase intention toward circular textile products

**DOI:** 10.3389/fpsyg.2022.1039435

**Published:** 2022-11-16

**Authors:** Imran Anwar, Mushahid Ali Shamsi, Sabiha Khatoon, Imran Saleem, Asiya Chaudhary

**Affiliations:** ^1^University Centre for Research and Development, Chandigarh University, Mohali, India; ^2^Department of Commerce, Aligarh Muslim University, Aligarh, India; ^3^Centre for Distance and Online Education (CDOE), Jamia Millia Islamia University, New Delhi, India; ^4^School of Business, University of Buraimi, Buraimi, Oman

**Keywords:** circular economy, circular textile products, purchase intention, environmental consciousness, textile waste management, sustainability

## Introduction

Prosperity creates extravagance. With economic development, rising consumer purchasing power, and expanding manufacturing sectors, demand for textiles and apparel is increasing daily. Unquestionably, it boosts national economies while producing unprecedented amounts of textile waste. The surge exacerbates environmental issues while also providing an opportunity to convert it into useful raw material through a circular economy (CE), a circular model in which virgin material use is reduced through reusing, recycling, and reproduction. Considering its significance, China and other Asian nations adopted this model long ago (Chen et al., 2021). However, it is still in its early stages in India, with only 1% of the material estimated to be recycled (Mehta, 2022). To reap the benefits from the circular model, the Indian textile and apparel industry must urgently transform into circular textile products (CTP), which require only a fifth of the materials and a seventh of the energy in their production compared to new products.

Furthermore, it facilitated businesses' participation in ecological actions such as raw material preservation and product value addition. It provides a viable route to the Indian apparel and textiles industry, which consumes an estimated 110 million tons of material per year and generates more than 90 million tons of fashion waste, which is disposed of in landfills or burned (Mehta, 2022). As a 2.3% contributor to GDP, 13% contributor to domestic manufacturing, and the fifth-largest apparel and textile exporter in the world (India Brand Equity Foundation, 2022), it is the responsibility of India's textile industry to shift from a linear paradigm (take, make, use, discard) to a circular model; it is also essential to encourage CTP in the domestic economy. However, the success of a company and its products depends on its customer base; thus, studies on the factors responsible for attracting and influencing consumers and their purchase intention/behavior for CTP are required in India.

Previous research by Singhal et al. (2019), Wang et al. (2020), and Kumar et al. (2021) demonstrated that several factors influence consumer intentions toward remanufactured products, with perceived risk and benefits being the most common influencing factors. Consumers who perceive some risks, such as financial, time, performance, social, and physical risks, are discouraged from making decisions (Wang and Hazen, 2015). In contrast, consumers who perceive benefits are encouraged to do so (Wahjudi et al., 2018) because they seek out personal benefits, such as discounts or incentives, low cost, subsidies, exchange value, and good quality, from the remanufactured products. Thus, the risk and benefits perception affects consumers' intentions and behavior. Moreover, previous research has shown that consumers' environmental consciousness (EC) plays an essential role in shaping their intentions for remanufactured, eco-friendly, and green products (Costa et al., 2021; Kumar et al., 2021) because one's knowledge or ignorance about an issue significantly impacts their consciousness and, consequently, decision-making process (Corral-Verdugo, 2022). Hence, integrating these variables in future studies adds to the body of knowledge on CE, particularly on consumer perceptions of CTP.

According to Ajzen (1991), the cognitive factors of the Theory of Planned Behavior (TPB) [viz. behavioral attitude (AT), social norms (SN), and perceived behavior control (PBC)] play a significant role in determining the behavioral intention of an individual. AT exhibits the mental state of a person's reflection (negative or positive) toward a particular behavior or intention (Paul et al., 2016). SN refers to the social effect that teachers, friends, family members, and others have on an individual to execute a particular behavior or decision (Bong Ko and Jin, 2017). PBC refers to the perceived ease (or difficulty) of executing any task that is vulnerable to an individual's psychological state (Khor and Hazen, 2016). It plays a vital role in forecasting his behavior and can modify intention owing to its direct influence (Wiederhold and Martinez, 2018). A resourceful person with a positive attitude, the support of family and friends, and the perception of ease has a strong desire for specific behavior. As a result, TPB has been widely applied across behavioral and intentional domains, establishing itself as the dominant expectancy-value theory (Shaw et al., 2000; Echegaray and Hansstein, 2017). In empirical studies, TPB has outperformed previous TRA in predictive ability (Beck and Ajzen, 1991; Giles and Cairns, 1995). TPB has also been used to investigate customers' purchase intention/behavior, perceptions, and willingness to pay for remanufactured, circular, green, and organic products. However, using TPB to judge intention for CTP is a new addition to the CE literature. Therefore, an empirical understanding of cognitive factors like EC, PB, PR, AT, SN, and PBC, and consumer purchase intentions in the context of CTP aids in filling a knowledge gap in the field, raising public awareness of CE and CTP, and luring businesspeople and the government to create laws and regulations that would encourage investment in CE-related infrastructure, technology, and education. As a result, small and medium-sized businesses would have new opportunities in India, and the circular business model would also create jobs, produce sustainable textiles, and preserve the environment. In order to gather primary data, this study uses questionnaires based on the above cognitive and psychological factors that will affect consumers' intention toward CTP.

## Materials and methods

The current study gathers the survey-based multivariate primary data on certain cognitive factors (viz., EC, PB, PR, AT, SN, and PBC) that influence Indian consumers' purchase intention toward CTP. The final data sample collected online through Google Forms comprised 409 respondents from Indian consumers (including 230 male and 179 female respondents). The data were first screened for missing, inappropriate, and outlier responses before sending them out to establish a measurement model (for reliability and validity assessment) using AMOS 24.0.

### Questionnaire development

The questionnaire was developed in two sections. The first section of the questionnaire was designed to ask about the demographic features of the respondents (viz., age, gender, education, and monthly income), awareness, and previous purchasing experience of CTPs (Yes/No). While the second section comprised seven latent variables (viz., environmental consciousness, perceived risks, perceived benefits, behavioral attitude, social norms, perceived behavioral control, and purchase intention). At the beginning of the questionnaire, the respondents were briefly introduced to the objectives of the study while informing them about the voluntariness of being a respondent in the survey. A seven-point Likert-type scale was used to capture the latent variables. To measure the latent variables, we borrowed validated scales from published sources. Measurement items (with their loadings and adoption sources) can be found in the appendix (see Appendix A).

### Participants, pilot study, and main survey

The study is focused on recruiting educated consumers from the National Capital Region (NCR) and Aligarh city of India. As the study is about assessing the nexus between environmental consciousness, knowledge of CTP, other cognitive variables, and purchase intention toward circular textile products, it is presumed that educated consumers might be more environmentally conscious and may have greater knowledge of CTPs. We adopted a bifold piloting approach for validating the questionnaire. In the first step, the questionnaire was examined by eight consumers and four consumer behavior and marketing researchers for the subjectivity and relevance of the measurement items used to measure the latent variables. After assimilating their suggestions and recommendations for altering the questionnaire, we advanced to a pilot survey taking a sample of 87 respondents from the NCR and Aligarh city. Upon checking the reliability statistics based on the pilot survey, we finalized the questionnaire and conducted the main survey in March 2022, adopting a systematic random mall-intercept sampling approach. We approached every fifth customer leaving the mall and asked them to participate in the survey while briefing them about the purpose and aims of the study. Those who agreed to participate in the survey were asked to scan a QR (code linked to an online Google Form questionnaire) for keeping anonymity and abiding by the social distancing precautionary norms. To every respondent, who scanned the QR code, we also offered a carry bag (a circular textile product) as a token of appreciation. Precisely, the survey lasted for 13 days (19th−31st March 2022), and about 600 consumers gave their consent and scanned the QR code for filling out the questionnaire. However, we obtained only 467 filled questionnaires until 31st March 2022.

### Data preparation

Before moving ahead with further statistical validation analysis (establishment of measurement model), we processed the data through a cleaning and screening process. Initially, we ensured that the data were not suffering from missing responses followed by unengaged responses. Upon evaluation, we found 16 missing cases and 23 unengaged responses and discarded them from the dataset. Further, we also checked for outlier responses applying Cook's distance approach and found that 19 responses were producing Cook's distance value over the threshold of 1 (Stevens, 2012); hence these 19 responses were also discarded from the dataset, and we achieved our final sample of 409 responses. Table 1 demonstrates the demographic features of the sample.

**Table 1:** Respondent's demographic profile (*N* = 409).

**Variable name**	**Category**	**Frequency**	**Percentage (%)**
Age	Less than 20 years	56	13.70
	20–30 years	210	51.30
	31–40 years	80	19.60
	Above 40 years	63	15.40
Gender	Male	230	56.20
	Female	179	43.70
Education	Up to intermediate	56	13.70
	Graduate	205	50.10
	Post-graduate and above	148	36.20
Monthly income	Below 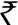 30,000	222	54.30
	Between 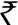 30,001–60,000	112	27.40
	Between 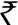 60,001–90,000	35	8.60
	Above 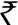 90,000	40	9.80
Awareness of CTP?	Yes	266	65.00
	No	143	35.00
Purchased CTP before?	Yes	147	35.90
	No	262	64.10

CTP, circular textile product.

## Results

### Measurement model: Fit indices, reliability, and validity

Post data cleaning and preparation process, we established the measurement model running a co-variance-based CFA model in AMOS 24.0. For achieving the model fit indices, standardized loadings, and average variance extracted (AVE) values (for convergence), we drew a CFA model taking all 25 observed items and allowing them to converge with their respective latent variables.

Table 2 confirms that the model fit indices are found to fall in the excellent category for each index parameter. The results from the CFA model (see Table 2) also affirm that observed items converge sufficiently (showing CFA loadings <0.70) with their respective latent variables, and consequently, AVE values for each latent variable are also found above the benchmark of 0.50 (Bagozzi and Yi, 1988; Hair et al., 2006) hence confirming that the data meet the criterion of convergence. Scale reliability was also assessed using composite reliability (CR) and Cronbach's alpha (α) statistics. Results from Table 2 confirm that each latent construct shows enough internal consistency with CR and α above the threshold of 0.70 (Bagozzi and Yi, 1988; Hair et al., 2006).

**Table 2:** CFA model fit Indices, Alpha, CR, and AVE.

**Model**	**CMIN/DF**	**GFI**	**TLI**	**CFI**	**RMSEA**
CFA Model	2.218	0.921	0.953	0.961	0.055
Recommended value	Acceptable 1-4	≥0.90	≥0.95	≥0.95	< 0.08
	Wheaton et al. (1977)	Shevlin and Miles (1998)	Hu and Bentler (1999)	Hu and Bentler (1999)	MacCallum et al. (1996)
**Variable name**	**No of items**	**Alpha (** ***α*** **)**	**CR**	**AVE**	
Purchase intention	4	0.856	0.855	0.596	
Behavioral attitude	3	0.835	0.834	0.626	
Social norms	4	0.812	0.794	0.597	
Perceived behavioral control	3	0.811	0.813	0.591	
Environmental consciousness	4	0.837	0.843	0.609	
Perceived risks	3	0.779	0.784	0.574	
Perceived benefits	4	0.851	0.854	0.593	

In addition to scale reliability and convergence, the measurement model also needs to fulfill the criterion of discriminant validity. As per Fornell and Larcker (1981) recommendation, each latent variable's squared root of AVE value (bold diagonal value in Table 3) must be greater than its bivariate correlations (off-diagonal values) with other latent variables. It is evident from Table 3 that bold diagonal values (squared root of AVE) for each variable are greater than below off-diagonal values hence meeting the criterion of discriminant validity. Table 3 also portrays descriptive statistics (viz., mean and SD) for each latent variable along with skewness statistics for assuming the multivariate normality. The data tends to possess multivariate normality if the skewness value ranges between −2 and +2 (Kline, 1998).

**Table 3:** Correlations, divergent validity, and descriptive statistics.

**Variable name**	**PI**	**AT**	**SN**	**PBC**	**EC**	**PR**	**PB**
PI	**0.772**						
AT	0.613**	**0.791**					
SN	0.591**	0.591**	**0.773**				
PBC	0.613**	0.649**	0.621**	**0.769**			
EC	0.655**	0.628**	0.499**	0.581**	**0.780**		
PR	−0.173**	−0.103*	−0.242**	−0.140**	−0.146**	**0.758**	
PB	0.336**	0.444**	0.468**	0.368**	0.353**	−0.474**	**0.770**
Mean	5.491	5.445	4.956	5.456	5.748	3.931	4.979
SD	1.127	1.066	1.233	1.182	1.098	1.409	1.324
Skewness	−0.680	−0.748	−0.591	−0.684	−0.227	0.199	−0.424

Standardized effects are significant at 1%, i.e., 1%.

PI, purchase intention; AT, behavioral attitude; SN, social norms; PBC, perceived behavioral control; EC, environmental consciousness; PR, perceived risks; PB, perceived benefits.

Correlations are significant at **1% and *5% levels.

## Significance and use of the data

The current study conceptualizes the causal effect of certain cognitive variables (viz., environmental consciousness, perceived risks, and perceived benefits) and the fundamental factors of the theory of planned behavior (TPB) (viz., behavioral attitude, social norms, and perceived behavioral control) on Indian consumers' purchase intention toward circular textile products (CTPs). This study provides a novel multivariate dataset in the research domain of consumers' behavior toward circular economy, circular textile products, and textile waste management, particularly in the Indian context. Due to the sheer scantiness of the literature on consumer behavior and CTPs in the Indian context, empirical studies are required to understand the role of cognitive factors like EC, PB, PR, AT, SN, and PBC in predicting consumer purchase intentions toward CTPs. These empirical researches would contribute to the knowledge gap in the field, raising public awareness of CE and CTPs, and attracting business people and the government to create laws and regulations to encourage investment in CE-related infrastructure, technology, and education. As a result, small and medium-sized businesses would have new opportunities in India, and the circular business model would also create jobs, produce sustainable textiles, and preserve the environment. The distinctiveness of the dataset is the inclusion of a cognitive environmental factor, i.e., consumers' environmental consciousness along with perceived risks and benefits as attributional variables CTPs, while adopting one of the most widely applied theories, the TPB, across behavioral and intentional domains. Given the dearth of literature and knowledge gap in the research domain of consumers' behavior toward circular economy, circular textile products, and textile waste management in the Indian context, the current dataset would be of great use for the researchers in understanding the role of factors like EC, PB, PR, AT, SN, and PBC in shaping consumers' purchase intentions toward CTPs. Moreover, the dataset may also be used by local authorities for knowing the consumers' perceived level of adopting CTPs thus enabling them to formulate the policies for promoting CTPs accordingly. The findings from this dataset would be beneficial for the government to raise public awareness of CE and CTPs and create laws and regulations to encourage investment in CE-related infrastructure, technology, and education. As the data are multivariate and possess metric and latent properties, they can be used for complex modeling like mediation and moderated-mediation. Moreover, comparable distribution of the data across the categories of gender, awareness, and prior experience of CTPs also offers the likelihood of testing a moderation or multigroup SEM model.

## Data availability statement

The datasets presented in this study can be found in online repositories. The names of the repository/repositories and accession number(s) can be found in the article/Supplementary material.

## Ethics statement

Written informed consent was obtained from the individual(s) for the publication of any potentially identifiable images or data included in this article.

## Author contributions

IA conceptualized the study, wrote the original draft, and performed the statistical analysis. MS developed the questionnaire and conducted the survey. SK performed the data curation, took care of the methodology, and wrote the second draft. IS and AC reviewed and edited the final draft. All authors contributed to the article and approved the submitted version.

## Conflict of interest

The authors declare that the research was conducted in the absence of any commercial or financial relationships that could be construed as a potential conflict of interest.

## Publisher's note

All claims expressed in this article are solely those of the authors and do not necessarily represent those of their affiliated organizations, or those of the publisher, the editors and the reviewers. Any product that may be evaluated in this article, or claim that may be made by its manufacturer, is not guaranteed or endorsed by the publisher.

## Appendix

**Appendix A:** Questionnaire items with their CFA loadings and source of adoption.

**Item code**	**Construct name and measurement item**	**CFA loading**
**Environmental consciousness**		
**(Source: Suki, 2016; Singhal et al., 2019)**		
EC1	“I am willing to make extraordinary efforts to purchase circular textile products to protect the environment.”	0.751
EC2	“Given a choice, I will prefer to purchase a circular textile product because it is less harmful to the environment.”	0.821
EC3	“I will purchase circular textile products because it contributes toward the sustainability of the environment.”	0.785
EC4	“I would prefer circular textile products over fresh textile products because it helps in limiting environmental pollution.”	0.765
**Perceived risks**		
**(Source: Wang et al., 2013)**		
PR1	“I am afraid that the frequent maintenance of circular textile products will waste my time and money.”	0.714
PR2	“I apprehend that circular textile products will have poor performance.”	0.827
PR3	“I fear that the use of circular textile products might lead to skin issues/problems.”	0.733
**Perceived benefits**		
**(Source: Singhal et al., 2019; Kumar et al., 2021)**		
PB1	“I will buy circular textile products because of their lower price.”	0.777
PB2	“I will purchase circular textile products because I will be able to buy more quantity of textile products at a low price.”	0.776
PB3	“I will purchase circular textile products because they will be available at a discount.”	0.816
PB4	“I will purchase circular textile products if I get an exchange offer in return for my used textiles.”	0.710
**Behavioral attitude**		
**(Source: Kumar et al., 2021)**		
AT1	“I would prefer buying circular textile products because they follow an eco-friendly process in manufacturing.”	0.810
AT2	“I would buy circular textile products because they are reproduced through circular production.”	0.735
AT3	“I would purchase circular textile products because it is an important idea regarding a sustainable environment.”	0.828
**Social norms**		
**(Source: Kumar et al., 2021)**		
SN1	“People with whom I get impressed influence me to purchase circular textile products.”	0.759
SN2	“The people important to me think I should purchase circular textile products.”	0.793
SN3	“My family and friends think purchasing circular textile products is a wise idea.”	0.725
SN4	“People who are important to me would approve of my decision to buy circular textile products.”	0.725
**Perceived behavioral control**		
**(Source: Kumar et al., 2021)**		
PBC1	“I will always try to purchase environmentally responsible circular textile products.”	0.747
PBC2	“I am confident that I will purchase circular textile products when I go for purchasing textile products.”	0.781
PBC3	“When I have the resources and opportunities, I will surely buy circular/remanufactured textile products.”	0.779
**Purchase Intention toward Circular Textile Products**		
**(Source: Singhal et al., 2019; Calvo-Porral and Lévy-Mangin, 2020)**		
PI1	“I will buy circular textile products in the future.”	0.801
PI2	“I am likely to buy circular textile products.”	0.741
PI3	“I will continue buying circular textile products.”	0.775
PI4	“I am excited to buy circular textile products.”	0.771
